# Incorporating gene co-expression network in identification of cancer prognosis markers

**DOI:** 10.1186/1471-2105-11-271

**Published:** 2010-05-20

**Authors:** Shuangge Ma, Mingyu Shi, Yang Li, Danhui Yi, Ben-Chang Shia

**Affiliations:** 1 School of Public Health, Yale University, New Haven, CT 06520, USA; 2 Clinical Epidemiology Research Center, VA CT Healthcare System, West Haven, CT 06516, USA; 3 Standard and Poor's, New York, NY 10041, USA; 4 School of Statistics, Renmin University, Beijing, China; 5 Department of Statistics and Information Science & Applied Statistics, Fu Jen Catholic University, Taipei Hsien R.O.C

## Abstract

**Background:**

Extensive biomedical studies have shown that clinical and environmental risk factors may not have sufficient predictive power for cancer prognosis. The development of high-throughput profiling technologies makes it possible to survey the whole genome and search for genomic markers with predictive power. Many existing studies assume the interchangeability of gene effects and ignore the coordination among them.

**Results:**

We adopt the weighted co-expression network to describe the interplay among genes. Although there are several different ways of defining gene networks, the weighted co-expression network may be preferred because of its computational simplicity, satisfactory empirical performance, and because it does not demand additional biological experiments. For cancer prognosis studies with gene expression measurements, we propose a new marker selection method that can properly incorporate the network connectivity of genes. We analyze six prognosis studies on breast cancer and lymphoma. We find that the proposed approach can identify genes that are significantly different from those using alternatives. We search published literature and find that genes identified using the proposed approach are biologically meaningful. In addition, they have better prediction performance and reproducibility than genes identified using alternatives.

**Conclusions:**

The network contains important information on the functionality of genes. Incorporating the network structure can improve cancer marker identification.

## Background

Cancer is a complex disease. Extensive biomedical studies have shown that clinical and environmental risk factors may not have sufficient predictive power for cancer prognosis. The development of high-throughput profiling technologies makes it possible to survey the whole genome and search for genomic markers that may have independent predictive power for cancer prognosis [1]. Gene signatures have been constructed for the prognosis of breast cancer, lymphoma, ovarian cancer, and many others [2]. In this article, we focus on gene expression data measured using microarrays but note that the proposed approach is also applicable to other profiling techniques.

Denote *T *as the cancer survival time and *C *as the censoring time. Denote *X *as the length-*d *gene expression measurements. Under right censoring, one observes (*Y *= *min*(*T*, *C*), Δ = *I*(*T *≤ *C*), *X*). In cancer genomic studies, the sample size *n *is much smaller than *d*. Dimension reduction or feature selection is needed along with model estimation [3-5]. Dimension reduction methods construct a small number of "super genes" using the linear combinations of all genes, whereas feature selection methods select a subset of important genes. Literature review suggests that performance of different methods is data-dependent, with no one dominating the other. The proposed method conducts feature selection. Since it is not the focus of this article, dimension reduction methods will not be further discussed.

Many existing methods assume the interchangeability of genes and ignore the interplay among them. Recent biomedical studies suggest that there is an inherent coordination among genes and, essentially, all biological functions of living cells are carried out through the coordinated effects of multiple genes. There are many ways of describing the interplay among genes. In this article, we focus on the gene network. In network analysis, nodes represent genes. Nodes are connected if the genes have similar biological functions and/or correlated expressions. There are subsets of nodes called "modules" that are tightly connected to each other. One way of defining the relative importance of a gene within a network is the connectivity, which measures how well this gene is connected with the rest of the genes. Highly connected genes have been referred to as "hub genes" and are more likely to have important biological functions.

In this article, we adopt the weighted co-expression network developed by Dr. Steve Horvath and his colleagues. We provide a brief description of the weighted co-expression network in the "Methods" section and refer to [6] for more details. The weighted co-expression network is built on the understanding that the coordinated co-expressions of genes encode interacting proteins with closely related biological functions and cellular processes [7]. Extensive studies have shown that modules in the weighted co-expression network usually have important biological implications. In addition, genes with higher connectivity are more likely to be involved in important molecular processes. Incorporating connectivity in the detection of differentially expressed genes can significantly improve reproducibility [8-14].

There are other ways of defining gene networks. Examples include the Boolean network, Bayesian network, use of continuous models and others. Compared with other networks, the weighted co-expression network may have the following advantages. First, it is computationally simple and can be easily constructed using existing software. Second, it does not require any additional biological experiments. And third, a large number of published studies have shown that it has satisfactory empirical performance. On the other hand, it may have certain drawbacks. The network is defined based on the correlations among gene expressions, which may not contain all of the information on the coordination of genes. In addition, the network construction is unsupervised and not tailored to any specific traits or disease outcomes.

In this article, for cancer prognosis studies with gene expression measurements, we construct the weighted co-expression network and measure the relative importance of genes using their connectivity. We develop a regularized gene selection method, which can effectively take the connectivity into consideration. We note that the proposed approach is relatively "independent" of the network construction and thus applicable with other networks. The proposed approach takes advantage of recent developments in regularized gene selection and network-based analysis but significantly advances from them along the following directions. Unlike existing methods that assume the interchangeability of genes, the proposed approach can account for the network structure and thus be more informative. This is partly reflected in the improved prediction performance and reproducibility. Unlike existing network analyses that focus on the detection of genes or modules marginally associated with phenotypes, the proposed approach can account for the joint effects of multiple genes. Cancer development and progression are caused by the joint effects of multiple gene mutations or defects as opposed to the disturbance of a single gene. Analysis that can account for the joint effects of multiple genes is more informative than analysis of marginal effects. An existing approach that can account for the joint effects of multiple genes is the eigengene-based approach. In eigengene analysis, principal component analysis is conducted on genes within the same modules. The first principal components are used in downstream analysis. Principal components use the linear combinations of all genes, which make the analysis results difficult to interpret. In addition, even in a module significantly associated with cancer prognosis, noisy genes may still exist, especially considering that the gene network is constructed in an unsupervised manner and not tailored to any specific cancer outcomes. The proposed approach conducts gene selection, can effectively remove noisy genes, and thus may have better interpretability.

## Results

### Data collection and processing

We collect six cancer prognosis studies with microarray measurements. We refer to them as data D1-D6 and provide brief descriptions below and in Table 1.

**Table 1:** Description of datasets.

Data	Disease	Platform	Gene	Sample
D1: Huang et al. (2003) [15]	Breast cancer	Affymetrix	12625	71
D2: Sotiriou et al. (2003) [16]	Breast cancer	cDNA	7650	98
D3: van't Veer et al. (2002) [17]	Breast cancer	Oligonucleotide	24481	78
D4: Sorlie et al. (2001) [18]	Breast cancer	cDNA	8102	58
D5: Rosenwald et al. (2003) [19]	MCL	cDNA	8810	92
D6: Rosenwald et al. (2002) [20]	DLBCL	cDNA	7399	240

Platform: platforms used for profiling; Gene: number of gene expressions measured; Sample: sample size.

#### D1

Breast cancer is the second leading cause of death from cancer among women in the United States. Despite major progress in breast cancer treatment, the ability to predict metastasis of the tumor remains limited. Huang et al. [15] reported a study investigating metastastic states and relapses in breast cancer patients. Affymetrix genechips were used for the profiling of 71 samples. Expression measurements on 12,625 probes were available.

#### D2

Sotiriou et al. [16] reported a study correlating gene expression measurements generated using cDNA with clinico-pathological characteristics and clinical outcomes in an unselected group of 99 node-negative and node-positive breast cancer patients. In the original analysis, the Cox model was used to identify genes marginally significantly associated with relapse-free survival. In this study, we analyze the 98 patients with complete survival information.

#### D3

Van't Veer et al. [17] reported a breast cancer prognosis study investigating the time to distant metastasis. 97 lymph node-negative breast cancer patients 55 years old or younger participated in this study. Among them, 46 developed distant metastases within 5 years. Complete information was available for 78 subjects. Expression levels of 24,481 gene probes were measured.

#### D4

The study reported in [18] was originally designed to classify breast carcinomas based on the variations in gene expression patterns. A total of 85 cDNA microarray experiments were conducted. This study showed that the cancers could be classified into a basal epithelial-like group, an ERBB2-overexpressing group, and a normal breast-like group. In our study, we analyze a subset of 58 patients who had survival information available.

#### D5

A study using microarray expression analysis of mantle cell lymphoma (MCL) was reported in [19]. Among 101 untreated patients with no history of previous lymphoma, 92 were classified as having MCL based on established morphologic and immunophenotypic criteria. Survival times of 64 patients were available, and the other 28 patients were censored. The median survival time was 2.8 years. Lymphochip DNA microarrays were used to quantify mRNA expressions in the lymphoma samples from the 92 patients. Gene expression data on 8,810 cDNA elements were available.

#### D6

Rosenwald et al. [20] reported a diffuse large B-cell lymphoma (DLBCL) prognosis study. This study retrospectively collected tumor biopsy specimens and clinical data for 240 patients with untreated DLBCL. The median follow up was 2.8 years, with 138 observed deaths. Lymphochip cDNA microarray was used to measure the expressions of 7,399 genes.

Among the six studies, four used cDNA, one used oligonucleotide arrays, and one used Affymetrix Genechips for profiling. We process each dataset separately as follows. We conduct microarray normalization using the lowess approach for cDNA data and the robust multi-array (RMA) approach for Affymetrix data. We impute missing measurements using the K-nearest neighbors approach. We select the 2,000 genes with the largest variances for downstream analysis. Since we expect the number of genes associated with cancer prognosis to be far less than 2,000, we conduct this prescreening to reduce computational cost. In addition, recent studies have shown that prescreening may improve gene selection accuracy [21]. We then normalize gene expressions to have zero median and unit variance.

### Construction of weighted co-expression network

We construct the weighted co-expression network using the approach described in the "Methods" section. For dataset D1-D6, 10, 10, 13, 12, 13, and 10 modules are constructed, respectively. More detailed results are available from the authors.

**Figure 1:**
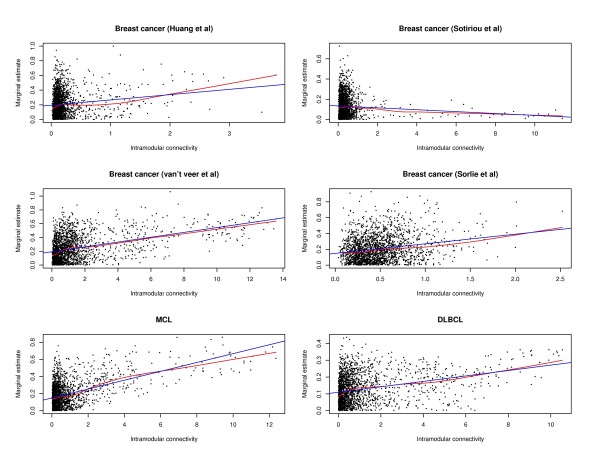
**Marginal gene significance (estimated regression coefficient from marginal Cox model) versus intramodular connectivity**. Red curve: lowess estimate; Blue straight line: linear fit.

In studies conducted by Dr. Horvath and his colleagues, it has been observed that the marginal gene significance and intramodular connectivity are positively correlated. For each gene, we fit a Cox model with the expression of this gene as the only covariate. The marginal gene significance is defined as the estimated regression coefficient from the marginal model. The intramodular connectivity is defined in the "Methods" section. Figure 1 shows that the marginal gene significance and intramodular connectivity are positively correlated for five of the datasets and (weakly) negatively correlated for one. Since the six datasets are "randomly" selected, we conclude that the positive correlation between marginal significance and intramodular connectivity tends to, but not necessarily, holds. We have experimented with other gene significance measures (for example, the log p-values from marginal models) and obtained similar results.

### Identification of prognosis markers

We apply the proposed approach and select the optimal tunings using five-fold cross validation. Table 2 shows that, for each dataset, a small number of genes are identified as prognosis markers. We provide more detailed information, including gene names and their estimated regression coefficients, in Additional File 1. We search NCBI and published literature for the biological implications of identified markers. We find that many identified genes have been previously identified as cancer markers. Details are provided in Additional File 2. Of note, although datasets D1-D4 are all on breast cancer, the corresponding studies had different settings (patient selection criteria, demographics, etc). Thus, we analyze them and interpret the identified markers separately.

**Table 2:** Data analysis results: gene identification and prediction.

	Gene identification			Prediction logrank	
Data	TGDR	Proposed	Overlap	TGDR	Proposed
D1	33	32	25	14.6	20.4
D2	15	10	7	4.1	11.2
D3	15	19	10	7.7	22.3
D4	19	20	13	11.4	19.3
D5	14	23	8	7.3	23.0
D6	26	27	20	13.9	28.4

A logrank statistic greater than 3.84 is significant at the 0.05 level.

### Evaluation

An important goal of cancer genomic studies is to construct predictive gene signatures. In addition, the evaluation of prediction performance can also provide indirect evaluation of the biological implications of genes. For the six datasets, we consider the following cross validation-based evaluation: (a) For *i *= 1 ... *n*, remove subject *i *from the data; (b) With the reduced data (with sample size *n *- 1), carry out cross validation and regularized estimation. Denote the estimated regression coefficient as  (we refer to the "Methods" section for the data and model setup); (c) Compute the predictive risk score  for the removed subject, where *X*_*i *_is the gene expression; (d) Repeat Steps (a)-(c) over all subjects; and (e) Dichotomize the predictive scores at the median. Create two risk groups. Compute the logrank statistic, which measures the difference of survival between the two groups [22]. Under the Null, the identified genes have no predictive power, and the logrank statistic is *χ*^2 ^distributed with degree of freedom 1. We show the predictive logrank statistics in Table 2. For all six datasets, the prediction performance is satisfactory, with p-values of the logrank statistics smaller than 0.05.

Beyond the prediction performance of all identified genes combined, we are also interested in the reproducibility of each identified gene. We consider the following approach: (a) Remove one subject from the data; (b) Conduct regularized estimation and gene selection using the reduced data with sample size *n *- 1; (c) Repeat Steps (a) and (b) over all subjects. For each gene, count *c*, the number of times it is identified in the *n *models. The ratio *c/n *provides a measure of reproducibility and is referred to as the "occurrence index" in the literature. Table 3 shows that the identified genes have occurrence indexes close to 1, which suggests very satisfactory reproducibility. In contrast, the genes not identified have much lower occurrence indexes.

**Table 3:** Data analysis results: reproducibility evaluation.

	TGDR		Proposed	
Data	Identified	Not identified	Identified	Not identified
D1	0.858 [0.437, 1]	0 [0, 0.479]	0.951 [0.479, 1]	0 [0, 0.409]
D2	0.919 [0.459, 1]	0 [0, 0.602]	0.954 [0.561, 1]	0 [0, 0.520]
D3	0.970 [0.567, 1]	0 [0, 0.340]	0.979 [0.804, 1]	0 [0, 0.175]
D4	0.910 [0.431, 1]	0 [0, 0.586]	0.931 [0.603, 1]	0 [0, 0.328]
D5	0.919 [0.489, 1]	0 [0, 0.446]	1 [0.620, 1]	0 [0, 0.326]
D6	0.958 [0.633, 1]	0 [0, 0.442]	1 [0.663, 1]	0 [0, 0.313]

Median [range] of the occurrence index.

### Analysis with TGDR

To further gauge performance of the proposed approach, we analyze the same data using the TGDR approach [23]. TGDR shares a similar thresholding regularization framework with the proposed approach. It has been shown to have satisfactory performance in several published studies. Unlike the proposed approach, it assumes the interchangeability of genes.

With TGDR, we also select the optimal tunings using five-fold cross validation. We show the estimation and cross validation-based prediction results in Table 2. TGDR also identifies a small number of genes. However, the gene sets identified by TGDR and the proposed approach can be significantly different. The TGDR prediction logrank statistics are considerably smaller than their counterparts under the proposed approach. We also evaluate the reproducibility and present the results in Table 3, showing that the occurrence indexes of TGDR-identified genes are smaller than those of the genes identified by the proposed approach.

## Discussion and Conclusions

In cancer studies, the construction of prognosis signatures from the analysis of high-throughput gene profiling studies is of great importance. In this article, we adopt the weighted co-expression network to describe the intercorrelation among genes. We adopt the intramodular connectivity as the measure of node importance. We propose a thresholding regularization gene selection approach, which directly accounts for the network structure and encourages the selection of genes with high intramodular connectivity. We analyze six prognosis studies on breast cancer and lymphoma. We find that the proposed approach can identify genes that are significantly different from those using TGDR. Genes identified using the proposed approach have sound biological bases, better prediction performance, and better reproducibility. As can be seen in the "Methods" section, the proposed approach is also applicable when other networks are adopted as long as a node importance measure like the intramodular connectivity can be defined. Investigating performance of the proposed approach under other networks and a systematic comparison of different networks are of interest but beyond the scope of this article.

For genes identified using the proposed approach, we search NCBI and published literature and find that many of them have sound biological bases and/or have been previously identified as breast cancer/lymphoma markers. This partly demonstrates the effectiveness of the proposed approach. There are also new discoveries that need to be further studied. We note that, although the identified signatures seem reasonable, extensive independent studies are needed to further validate those signatures before they can be used in clinical practice. We conduct prediction evaluation and find that the logrank statistics are highly significant for all six datasets and larger than those under TGDR. One of the ultimate goals of genomic studies is to improve prediction performance and reliability of disease signatures. The improvement observed in this study, although may not be dramatic, can still be valuable.

The proposed approach shares a similar thresholding regularization framework with TGDR and other thresholding methods. However, it differs significantly from existing approaches by accounting for the network structure. Conceptually, the selection is conducted with respect to both gradient (which measures gene importance in statistical models) and connectivity (which measures gene importance in networks). The proposed approach enjoys the advantages of TGDR (for example, low computational cost) and has better empirical performance. This study can be extended in several directions. First, it may be interesting to investigate the performance of the proposed approach under other networks and/or using other node importance measures. Second, gene signatures identified in this study may be compared with those using other methods and/or in other studies. Third, there are many gene selection approaches that ignore the interplay among genes. It may be possible to follow a similar strategy and extend them to incorporate the network structure. Lastly, it may be possible to consider other ways of incorporating gene intercorrelation using, for example, the pathway structure.

## Methods

The proposed analysis consists of the following steps. First, we construct statistical models linking gene expressions with cancer survival outcomes. Second, we construct the weighted co-expression network. The rationale of the network construction and its pros and cons have been discussed in detail elsewhere [6]. We briefly discuss them for the integrity of this article. The proposed approach is a thresholding regularized, iterative approach. It uses gradient thresholding, can be much more effective than the simple thresholding, and significantly advances from other thresholding approaches by accounting for the network structure.

### Statistical modeling

We assume that the *d *gene expressions are associated with cancer survival through the Cox proportional hazards model [22], where the conditional hazard function *λ *(*t*|*X*) = *λ*_0_(*t*) exp (*β' X*). Here, *λ*_0_(*t*) is the unknown baseline hazard, and *β *is the length-*d *regression coefficient. We assume *n *iid copies of (*Y*, Δ, *X*): (*Y*_*i*_, *δ*_*i*_, *X*_*i*_); *i *= 1 ... *n*. Denote *r*_*i *_= {*k*: *Y*_*k*_≥ *Y*_*i*_} as the at-risk set at time *Y*_*i*_. The log-partial likelihood function is 

### Construction of weighted co-expression network

Construction of the weighted co-expression network consists of the following steps.

1. For genes *k *and *j *(= 1 ... *d*), compute *cor*(*k*, *j*), the Pearson correlation coefficient of their expressions. Compute the similarity measure *S*(*k*, *j*) = |*cor*(*k*, *j*)|;

2. Compute the adjacency function *a*_*k*, *j *_= *S*^*b *^(*k*, *j*), where the adjacency parameter *b *is chosen using the scale-free topology criterion;

3. For gene *k*, compute its connectivity ;

4. For gene *k *(= 1 ... *d*), compute the topological overlap based dissimilarity measure *d*_*k*, *j *_= 1 - *ω*_*k*, *j *_where *ω*_*k*, *j *_= (*l*_*k*, *j *_+ *a*_*k*, *j*_)/(*min *(*C*_*k*_, *C*_*j*_) + 1 - *a*_*k*, *j*_) and . Define the dissimilarity matrix *D*, whose (*k*, *j*)th element is *d*_*k*_, _*j*_;

5. Identify network modules using matrix D and the hierarchical clustering approach. Apply the dynamic tree cut approach [24] to cut the clustering tree (dendrogram), and identify the resulting branches as modules;

6. For gene *k*, compute its intramodular connectivity , which is defined in a similar manner as in Step 3. The difference is that the sum is over genes within the same module.

In Steps 1 and 2, we define the adjacency measure between genes using the power transformation of correlation coefficients. We adopt the weighted network, which can measure not only whether two genes are connected but also the connection strength. The power adjacency function has the attractive factorization property. We find that, for the six datasets analyzed, *b *= 6, which has been suggested in several published studies, can lead to results satisfying the scale-free topology criterion. In Step 4, we use the topological overlap dissimilarity measure [25], which has been found to result in biologically meaningful modules. Here, modules are defined as sets of highly connected genes. The advantage of the dynamic tree cut approach in Step 5 has been discussed in [24]. We compute the intramodular connectivity in Step 6, which can be more meaningful than the whole-network connectivity.

### Network based gene selection

The proposed approach uses thresholding regularization to discriminate important genes from noisy ones. The basic strategy is similar to that of simple thresholding [26]. We first compute an importance measurement for each gene. We then set a data-dependent threshold. Genes with importance measures larger than the threshold are identified, whereas genes with importance measures smaller than the threshold are screened out. Only estimates for those identified genes are updated. The "computing (significance), thresholding, and updating" process iterates until a certain stopping criterion is reached. In gene selection, we incorporate the network structure and encourage the selection of genes with higher intramodular connectivity. This strategy has been motivated by the observation that intramodular connectivity tends to be correlated with gene significance in [8,11,14] as well as Figure 1.

Let Δ*ν *be the small positive increment. In the numerical implementation, we set Δ*ν *= 10^-3^. Denote 0 ≤ *τ*_1_, *τ*_2 _≤ 1 as the thresholds for gradient and connectivity, respectively. The proposed approach consists of the following steps.

1. Initialize *β *= 0;

2. With the current estimate of *β*, compute the length *d *vector of gradient *g *= ∂ *R*_*n*_/∂*β*. Denote *g*_*i *_as the *i*th component of *g*;

3. Compute the length-*d gradient thresholding vector f*^1^, where its *i*th component is 

4. Compute the length-*d connectivity thresholding vector f*^2^, where its *i*th component is 

5. Update the estimate 

6. Iterate Steps 2-5 *K *times, where *K *is determined via cross-validation.

As with many other gene selection methods, the above algorithm starts with no gene identified (Step 1). In Step 2, we compute the gradients, which measure the increase in likelihood if the corresponding estimates are updated. Thus, the gradients can measure the relative importance of genes in maximizing the likelihood function. Particularly, it is expected that more important genes tend to have larger gradients. As one reviewer pointed out, it is possible to use a more formal importance measure (for example, for a hypothesis testing). However, we note that, such a measure may not be directly related to maximization of likelihood, can be computationally expensive, and hence is not pursued. In Steps 3 and 4, thresholding regularization is used to select important genes. More specifically, in Step 3, we compare the gradient of a gene with those of the others. This step of thresholding can remove unimportant genes with small gradients. In Step 4, for genes with large gradients, we add the second level of thresholding and select those with large connectivity. By combining the two thresholding vectors in Step 5, we select genes with not only large gradients but also high connectivity. Only estimates of those selected genes are updated.

The proposed approach involves three tuning parameters. Their effects on the regularized estimates are as follows. When *K *is fixed, the values of the thresholds affect the level of sparsity. Particularly, when the thresholds decrease, the number of genes (identified as important) increases. When the thresholds are fixed and *K *increases, the number of genes (identified as important) increases.

We select the values of *K *and thresholds using V-fold cross validation, which can provide partial protection against overfitting. In our data analysis, we find that the cross validation-selected tunings can effectively balance between the goodness-of-fit and model sparsity. Our limited experiences suggest that the proposed approach has satisfactory convergence properties and stability.

## Authors' contributions

All authors were involved in the study design and writing. SM conducted the data analysis. All authors read and approved the final manuscript.

## Supplementary Material

Additional file 1 **Data analysis results**. This Excel file contains information on the genes identified using the proposed approach and the estimated regression coefficients. Click here for file

Additional file 2 **Detailed information on the identified genes**. This .pdf file contains detailed information on the biological implications of the identified prognosis markers. Click here for file
