# Supplementary material for: Incorporating gene co-expression network in identification of cancer prognosis markers
Source: BMC Bioinformatics. 2010 May 20;11:271. doi: 10.1186/1471-2105-11-271 (PMC2881088; doi:10.1186/1471-2105-11-271)
Supplement: Additional file 2 — Detailed information on the identified genes. This .pdf file contains detailed information on the biological implications of the identified prognosis markers. [file 1471-2105-11-271-S2.PDF]

Incorporating gene co-expression network in identification of cancer prognosis markers

By Shuangge Ma, Mingyu Shi, Yang Li, Danhui Yi, Ben-Chang Shia

Prognosis markers identified

Dataset D1. We find that many identified genes have important biological implications. Specifically, the protein encoded by gene MCM4 is one of the highly conserved mini-chromosome maintenance proteins (MCM) that are essential for the initiation of eukaryotic genome replication and cell growth. In mouse models, mutation of this gene increases the rate of flawed copies of genetic material, leading to breast cancers. Gene CTBP2 produces alternative transcripts encoding two distinct proteins. One protein is a transcriptional repressor, while the other isoform is a major component of specialized synapses known as synaptic riobbons. It is inferred to be associated with breast cancer via Fenretinide, Fluorouracil, Genisterin, and Tretinoin [1]. Gene PPIF is associated with endometrial cancer and lung cancer. It is inferred to be associated with breast cancer via Curcumin, Fenretinide, Genistein, and Progesterone [1–3]. ACLY is the first key enzyme of lipogenesis and a crosslink between glucose and lipid metabolism. ACLY overexpression or activation have been reported in breast, urinary bladder, liver, stomach, colon, prostate, and lung tumors. ATP citrate lyase inhibition can suppress tumor cell growth [4]. The protein encoded by gene PTP4A1 belongs to a small class of prenylated protein tyrosine phosphatases (PTPs). PTPs are cell signaling molecules that play regulatory roles in a variety of cellular processes. Overexpression of this gene in mammalian cells confers a transformed phenotype, which implicates its role in tumorigenesis. Overexpression of gene TACSTD2 is associated with a higher risk and/or poor prognosis of colon cancer, pancreatic cancer, oral squamous cell carcinoma, and colorectal cancer, which suggests its essential role in cancer development and progression. In both mouse mammary gland and human breast, the family of Iroquois-class (IRX) homeobox genes play important roles in development and neoplasia [5]. Gene EIF2AK2 is overexpressed in breast cancer cells compared to normal breast epithelial cells [6]. Gene SART1 encodes two proteins, the SART1(800) protein expressed in the nucleus of the majority of proliferating cells and the SART1(259) protein expressed in cancers. Kawamoto et al. investigated expression of the SART1 gene encoding tumor rejection antigens in breast cancer patients. SART1 mRNA was found to be expressed in all of the samples tested [7]. Gene RPS6KA2 encodes a member of the RSK (ribosomal S6 kinase) family of serine/threonine kinases. This kinase contains two non-identical kinase catalytic domains and phosphorylates various substrates, including members of the mitogen-activated kinase (MAPK) signalling pathway. The activity of this protein has been implicated in controlling cell

growth and differentiation. This gene is a tumor suppressor gene for ovarian cancer, which has a genomic basis similar to that of breast cancer [8]. Overexpression of ZNF146 in tumors may alter the balance between hRap1 and other telomeric proteins. Thus, the function of ZNF146 may be linked to telomere regulation. This gene is one of the breast cancer markers identified in [9]. It has been shown that knockdown of gene RPS14 (resulting in $\sim 60\%$ reduction in protein expression) significantly recapitulates the phenotypes of $5q^{-}$ syndrome, which is a myelodysplastic syndrome subtype associated with an increased risk of developing cancer [10]. The LIM domain only protein 7 (LMO7) is a member of the PDZ and LIM domain-containing protein family and a candidate gene with possible roles in embryonic development and breast cancer progression [11]. Gene NOLC1 is essential for TP53 to regulate MDM2 expression, which plays a key role in breast cancer development through mediating cell response to various stresses [12]. The protein encoded by gene CSNK1E is a serine/threonine protein kinase and a member of the casein kinase I protein family, whose members have been implicated in DNA replication and repair. Kim et al. identified this gene as required for the proliferation of breast cancer cells [13]. Gene NOTCH2 encodes a member of the Notch family. Notch family members play a role in a variety of developmental processes by controlling cell fate decisions. Florena et al. established the association between NOTCH2 expression and invasive human breast cancer and suggested that this gene may also represent new appealing therapeutic targets [14]. ITGAE is integrin, alpha E. These cell-surface adhesion molecules are known to play a major role in diverse cellular and developmental processes including morphogenesis, hemostasis, leukocyte activation, cellular adhesion, and homing. Clark et al. identified gene ITGAE as a marker for breast cancer [15]. The protein encoded by gene PTPRG is a member of the protein tyrosine phosphatase (PTP) family. PTPs are signaling molecules that regulate a variety of cellular processes including cell growth, differentiation, mitotic cycle, and oncogenic transformation. Gene DLX5 is crucial for embryonic development. It promotes cancer by activating the expression of oncogene MYC. In addition, since gene DLX5 is inactive in normal adults, it can be an ideal target for anti-breast cancer drugs.

Dataset D2. We find that the genes identified in dataset D2 also have sound biological basis. UBR2 is one of the general angiogenesis genes and is involved in protein regulation. Gene MCM4 is one of the six related MCM genes that play essential roles in replications. Those genes have remained largely unchanged throughout evolution and are evident in archaebacteria, yeast, mammals, and more. In mouse models, Schimenti and colleagues found that impaired MCM4 genes led exclusively to breast cancer. Gene KIAA0090 is one of the breast cancer markers identified in [16]. Mutations in the WAS gene cause the

Wiskott-Aldrich syndrome. In addition, they lead to the production of an abnormally short, nonfunctional version of WASP or prevent cells from producing any WASP at all. Mutations that entirely prevent the production of WASP tend to be associated with more severe symptoms and a greater risk of developing cancer [17]. Cell division cycle 7 is a widely expressed protein kinase implicated in cell division, cell cycle checkpoint mechanisms, and cancer progression. Choschzick et al. found that there was a tendency towards worse prognosis in cell division cycle 7 positive as compared to negative breast cancers. Cell division cycle 7 is a replication associated protein with relationships to gene amplification and genomic instability in breast carcinomas [18]. Gene IRF5 encodes a member of the interferon regulatory factor (IRF) family, a group of transcription factors with diverse roles including virus-mediated activation of interferon and modulation of cell growth, differentiation, apoptosis, and immune system activity. Its implication in breast cancer has been discussed in Takaoka et al [19]. The protein encoded by gene FKBP1A is a member of the immunophilin protein family, which play a role in immunoregulation and basic cellular processes involving protein folding and trafficking. The FKBP gene family play an important role in antitumor activities [20]. Gene YWHAZ encodes proteins belonging to the 14-3-3 family of proteins which mediate signal transduction by binding to phosphoserine-containing proteins. It cooperates with ErbB2 to promote ductal carcinoma in situ progression to invasive breast cancer by inducing epithelial-mesenchymal transition [21]. Gene SLC25A5 is a member of the mitochondrial carrier subfamily of solute carrier protein genes. SLC25A5-specific RNA interference approach to inhibit SLC25A5 expression can result in breast cancer cell growth arrest [22]. The protein encoded by gene MADD is a death domain-containing adaptor protein that interacts with the death domain of TNF-alpha receptor 1 to activate mitogen-activated protein kinase (MAPK) and propagate the apoptotic signal. This gene is intimately involved in anti-apoptotic and cell survival processes in cancer cells.

Dataset D3. Among genes identified in dataset D3, gene MSRA resides on chromosome 8p and is a candidate metastasis suppressor. A deletion of chromosome 8p plays an important role in the tumor progression and metastasis of many kinds of malignancies including colorectal, bladder, breast, larynx, renal, and lung cancers [23]. Molecular profiling of the NCI-60 cancer cell lines using five different microarray platforms shows strong negative correlations of ASNA expression and DNC copy number with sensitivity of I-ASP in multiple cancers including leukemia, ovarian cancer, and breast cancer. CENPA encodes a centromere protein which contains a histone H3 related histone fold domain that is required for targeting to the centromere. It has been found to predict clinical outcomes in early stage breast

cancer [24]. Gene PTTG3P is inferred to be associated with breast cancer via Fluorouracil [25]. SLC2A12 belongs to a family of transporters that catalyze the uptake of sugars through facilitated diffusion. It is one of the breast cancer markers identified in [26]. Ajiro et al. reported that gene-expression profiling analysis of breast cancer cells, semi-quantitative RT-PCR, Northern blotting, and Western blotting confirmed RQCD1 to be frequently up-regulated in breast cancer specimens and breast cancer cell lines. On the other hand, its expression was very weak or hardly detectable in normal human tissues except testis, indicating this molecule to be a novel cancer-testis antigen [27]. Cyclin-dependent kinases (CDK) belong to a group of protein kinases involved in the regulation of cell cycle and transcription and mRNA processing. Studies have shown that in cell culture a selective CDK inhibitor is preferentially effective in ER positive disease and acts synergistically with tamoxifen or trastuzumab [28]. In a recent study, Cheng et al. [29] showed that SCUBE2 was mainly expressed in vascular endothelial and mammary ductal epithelial cells in normal breast tissue. There is a positive staining for SCUBE2 in 55% of primary breast tumors. Patients with positive SCUBE2 protein-expressing tumors have better prognosis than those with negative SCUBE2 protein-expressing tumors in terms of disease-free survival. Gene KIF20A is inferred to be associated with breast cancer through Doxorubicin, Estradiol, Genistein, Progesterone, Raloxifene, and bisphenol A [1]. Overexpression of gene NMU (neuromedin U) has been found in multiple cancers including pancreatic cancer and lung cancer, which suggests its potential essential role in the growth and progression of cancer cells. Gene AGBP1 is one of the breast cancer markers identified in [30]. Gene RAB6B belongs to the RAS oncogene family, which play an important role in breast cancer development. Gene DIAPH3 is involved in cell adhesion, cell motility, and cytoskeleton organization. It is one of the genes in the 70-gene breast cancer signature. A recent study shows that gene HJURP (Holliday junction recognition protein) is expressed at a higher level in breast cancer than in normal breast tissues. In addition, HJURP mRNA levels are significantly associated with ER status, PR status, SBR grade, and Ki67 proliferation indices. Higher HJURP mRNA levels significantly decrease disease-free and overall survival [31]. Studies have found differential expression of gene CIRBP (cold inducible RNA binding protein) in breast carcinoma. In addition, this gene is inferred to be associated with breast cancer via Tamoxifen.

Dataset D4. Among genes identified in dataset D4, gene CACNA2D2 is identified in the homozygous deletion region of chromosome 3p21.3 in human lung and breast cancers. Expression deficiency of the CACNA2D2 in cancer cells suggests a possible link of it to Ca2+ signaling in the pathogenesis of lung and breast cancers [32]. Gene HECW1 functionally interacts with p53 and contributes to the induction of

apoptosis in cancerous cells bearing wild-type p53. Gene ADD2 encodes protein ADDB, which promotes the assembly of the spectrin-actin network. It is one of the breast cancer markers listed in [33]. ANG is an angiogenesis gene. Elevated expression of this gene is associated with prostate cancer, colorectal cancer, and breast cancer. Gene MET may play a critical role in the development of the most aggressive breast cancers and may be a rational therapeutic target. The proto-oncogene MET product is the hepatocyte growth factor receptor and encodes tyrosine-kinase activity [34]. The protein encoded by CSTA is one of the precursor proteins of cornified cell envelope in keratinocytes and plays a role in epidermal development and maintenance. Stefins have been proposed as prognostic and diagnostic tools for cancer. Particularly, Stefin A expression reduces distant metastatis in breast cancer, which may be due to the inhibition of cysteine cathepsins [35]. Gene ARNT2 encodes a member of the basic-helix-loop-helix-Per-Arnt-Sim (bHLH-PAS) superfamily of transcription factors. A recent study suggests the possible use of ARNT2 as a breast cancer prognostic marker [36]. Gene PTDSS1 resides in the 8q22 region of chromosome 8, together with genes MTDH and LAPTM4. These genes have been identified associated with poor prognosis in breast cancer, which may point to a new therapeutic target. Gene ITPR3 is inferred to be associated with breast cancer through its connection with Raloxifene and Tamoxifen [1]. Gene SLC25A5 is a member of the mitochondrial carrier subfamily of solute carrier protein genes. SLC25A5-specific RNA interference approach to inhibit SLC25A5 expression can result in breast cancer cell growth arrest [22]. Gene BCL2 encodes an integral outer mitochondrial membrane protein that blocks the apoptotic death of some cells such as lymphocytes. It is related to good prognosis of breast cancer. In addition, p53(+)BCL2(-) phenotype is significantly correlated with the basal cell-like subtype and may be associated with the biologic aggressiveness of this cohort of molecular breast cancer. BCL2 expression is highly associated with an increased risk of local recurrence in patients with early stage breast cancer. Inhibition of system L (SLC3A2) reduces the growth of cultured human breast cancer cells. The protein encoded by gene CCNE1 functions as a regulator of CDK kinases. Overexpression of this gene has been observed in many tumors, which results in chromosome instability and thus may contribute to tumorigenesis. Particularly, overexpression of CCNE1 has been associated with breast cancer invasiveness. In addition, it has been found that CCNE1 has distinct expressions in different breast cancer subtypes. It qualifies as an independent prognostic marker for lymph node-negative breast cancer patients. Expression of CCNE1 in breast cancer cells is associated with negative steroid receptor status, HER2 presence, higher tumor grade,

and higher proliferation index.

Dataset D5. We search literature and find the following biological implications of genes identified in dataset D5. CKS1B protein binds to the catalytic subunit of the cyclin dependent kinases and is essential for their biological function. The expression of CKS1B is elevated in multiple cancers, including breast cancer, lymphoma, myeloma, colon cancer, prostate cancer, lung cancer, renal carcinoma, oesophageal squamous cell carcinoma, salivary cancer, serous ovarian cancer, bladder cancer, and urothelial carcinoma. In addition, it is also associated with poor prognosis in multiple cancers, including myeloma, breast cancer, lymphoma, renal carcinoma, and ovarian cancer. Gene PINK1 (Serine/threonine kinase 6) encodes a serine/threonine protein kinase that localizes to mitochondria. It protects cells from stress-induced mitochondrial dysfunction. The protein encoded by gene CEBPB is important in the regulation of genes involved in immune and inflammatory responses and has been shown to bind to the IL-1 response element in the IL-6 gene as well as to regulatory regions of several acute-phase and cytokine genes. It is one of the lymphoma markers identified in [37]. Gene BIRC5 (baculoviral IAP repeat-containing 5) is a member of the inhibitor of apoptosis (IAP) gene family, which encode negative regulatory proteins that prevent apoptotic cell death. Expression of this gene has been found associated with outcomes of DLBCL and its subtypes [38, 39]. Gene SOCS1 (Suppressor of cytokine signaling 1) encodes a member of the STAT induced STAT inhibitor (SSI), also known as suppressor of cytokine signaling (SOCS), family. It has been found inactive in primary mediastinal large B-cell lymphoma, HL, diffuse large B-cell lymphoma, and follicular lymphoma. Mutations of this gene have been identified as a tumor suppressor in mediastinal b-cell lymphoma. Gene SOCS-1 triggers an oncogenic pathway which is operative in both Hodgkin and non-Hodgkin lymphomas. Gene UBE2S belongs to the Ubiquitin mediated proteolysis pathway, which is activated in DLBCL cell lines by doxorubicin and identified as a marker [40]. Flap endonuclease 1 (Fen1) promotes tumor progression. In mouse models, it is found that mutations in Fen1 are associated with early-onset lymphomas and extensive embryonic apoptosis [41]. Gene SLC25A5 (Solute carrier family 25 (mitochondrial carrier; adenine nucleotide translocator), member 5) is a member of the mitochondrial carrier subfamily of solute carrier protein genes. Alterations in expressions of this gene are associated with the transformation of follicular lymphoma to DLBCL [42]. Gene HMGA1 (high mobility group AT-hook 1) encodes a non-histone protein involved in many cellular processes, including regulation of inducible gene transcription, integration of retroviruses into chromosomes, and the metastatic progression of cancer cells. Blocking HMGA1 function in human leukemia or lymphoma cells can lead to decreased cellular motility

and foci formation. CDC20 appears to act as a regulatory protein interacting with several other proteins at multiple points in the cell cycle. It is frequently upregulated in many types of malignancies. It is found that p53 inhibits tumor cell growth through the indirect regulation of CDC20 and that CDC20 might be a good therapeutic target for a broad spectrum of human cancer [43]. Gene CENPF (Centromere protein F, 350/400ka (mitosin)) belongs to the cell cycle mitotic pathway. This gene encodes a protein that is associated with the centromere-kinetochore complex. It is identified to be associated with unfavorable outcomes in advanced Hodgkin lymphoma. It is also associated with the transformation of follicular lymphoma to DLBCL [44]. The protein encoded by gene PCNA (proliferating cell nuclear antigen) is found in the nucleus and is a cofactor of DNA polymerase delta. In response to DNA damage, this protein is ubiquitinated and involved in the RAD6-dependent DNA repair pathway. Gene PCNA has been found overexpressed in pancreatic cancer, colon cancer, colorectal cancer, prostatic cancer, pancreatic cancer, lung cancer, and cervix cancer. It has been identified to be associated with non-Hodgkin lymphomas and childhood lymphomas [45, 46]. Gene HHEX (Hematopoietically expressed homeobox) encodes a member of the homeobox family of transcription factors, many of which are involved in developmental processes. It controls cell survival through coordinate transcriptional regulation of VEGF signaling. It has been identified as a marker of myoloid leukemia, which shares a similar genomic basis with lymphoma [47]. Gene RNF2 (Huntingtin interacting protein 1, also known as ring finger protein 2) encodes one of the PcG proteins, which form the multiprotein complexes that are important for the transcription repression of various genes involved in development and cell proliferation. Gene NPM3 has been identified as a marker for survival in lymphoma patients [48].

Dataset D6. We find the following biological implications of genes identified in dataset D6. Uridine kinase-like 1 mRNA is most abundant in tumor cell lines, indicating that it may be important in tumor cell growth and/or survival. The protein encoded by gene CDK7 is a member of the cyclin-dependent protein kinase (CDK) family. CDK family members are known to be important regulators of cell cycle progression. It has been identified as a marker for leukemia and lymphoma, but not for solid tumors [49]. Gene DNTTIP2 interacts with estrogen receptor alpha and meditates sodium selenite, which has an important implication in B cell lymphoma [50]. Gene SEPT1 is a member of the septin family of GTPases, which have been implicated in the pathogenesis of ovarian cancer and murine T-cell lymphoma. Our study suggests that it may also be associated with DLBCL. Lee et al. suggested that gene C14Orf1 (chromosome 14 open reading frame 1) had not been functionally characterized in mammalian cells and was poorly

annotated. However, it is believed that this gene plays a role in sterol biosynthesis, which has important implications in multiple cancers including lymphoma [51]. Bone morphogenetic proteins (BMP), belonging to the transforming growth factor-beta superfamily, are important regulators of cell growth, differentiation, and apoptosis. BMP-6 inhibits growth of mature human B cells. It has an important effect on malignant lymphoma, although the biological mechanisms remain unclear [52]. HLA-DQA1 belongs to the HLA class II alpha chain paralogues. The class II molecule is a heterodimer consisting of an alpha (DQA) and a beta chain (DQB), both anchored in the membrane. It plays a central role in the immune system by presenting peptides derived from extracellular proteins. Genes HLA-DRA and HLA-DRB have biological functions similar to those of HLA-DQA. GRO2 is an oncogene and has been identified as a marker for T cell lymphoma. Our study suggests its potential role in DLBCL [53]. Gene CCL13 is one of several Cys-Cys (CC) cytokine genes clustered on the q-arm of chromosome 17. Cytokines are a family of secreted proteins involved in immunoregulatory and inflammatory processes. The cytokine encoded by this gene displays chemotactic activity for monocytes, lymphocytes, basophils, and eosinophils. This chemokine plays a role in accumulation of leukocytes during inflammation. The protein encoded by gene RAC1 is a GTPase which belongs to the RAS superfamily of small GTP-binding proteins. Members of this superfamily appear to regulate a diverse array of cellular events, including the control of cell growth, cytoskeletal reorganization, and the activation of protein kinases. Gene POSTN (osteoblast specific factor 2 (fasciclin I-like)) has been found to be overexpressed in non-small-cell lung cancer, colon cancer, bladder cancer, gastric cancer, and lymph node metastasis, which suggests its fundamental role in cancer development. Gene SPARC (secreted protein, acidic, cysteine-rich (osteonectin)) has also been identified to be associated with multiple cancers including gastric cancer, ovarian cancer, lung cancer, breast cancer, pancreatic cancer, and prostate cancer.

References

1. **Comparative Toxigenomics Database** [http://ctd.mdibl.org/].
2. Paulssen RH, Moe B, Gronaas H, Orbo A: **Gene expression in endometrial cancer cells (Ishikaw) after short time high dose exposure to progesterone**. *Steroids* 2008, **73**:116–128.
3. Labbozzetta M, Norarbartolo M, Poma P, Maurici A, Inguglia L, Marchetti P, Rizii M, Baruchello R, Simoni D, D'Alessandro N: **Curcumin as a possible lead compound against hormone-independent, multidrug-resistant breast cancer**. *Annals of the New York Academy of Sciences* 2009, **1155**:278–283.
4. Yancy HF, Mason JA, Peters S, Thompson CE, Littleton GK, Jett M, Day AA: **Metastatic progression and gene expression between breast cancer cell lines from African American and Caucasian women**. *Journal of Carcinogenesis* 2007, **6**:8.
5. Lewis MT, Ross S, Strickland PA, Snyder CJ, Daniel CW: **Regulated expression patterns of IRX-2, an Iroquois-class homeobox gene, in the human breast**. *Cell and Tissue Research* 1999, **296**:549–554.
6. Kim SH, Forman AP, Mathews MB, Gunnery S: **Human breast cancer cells contain elevanted levels and activity of the protein kinase PKR**. *Oncogene* 2000, **19**:3086–3094.

7. Kawamoto M, Shichijo S, Imai Y, Imaizumi T, Koga T, Yanaga H, Itoh K: **Expression of the SART-1 tumor rejection antigen in breast cancer**. *International Journal of Cancer* 1999, **80**:64–67.
8. Bignone PA, Lee KY, Liu Y, Emilion G, Finch J, Soosay AER, Charnock FML, Beck S, Dunham I, Mungall AJ, Ganesan TS: **RPS6KA2, a putative tumour suppressor gene at 6q27 in sporadic epithelial ovarian cancer**. *Oncogene* 2007, **26**:683–700.
9. Cleator SJ, Powles TJ, Dexter T, Fulford L, Mackay A, Smith IE, Valgeirsson H, Ashworth A, Dowsett M: **The effect of the stromal component of breast tumours on prediction of clinical outcome using gene expression microarray analysis**. *Breast Cancer Research* 2006, **8**:R32.
10. Alderton GK: **Tumour suppressors: one-hit wonder**. *Nature Review Cancer* 2008, **8**:162–163.
11. Ott EB, van den Akker NM, Sakalis PA, Gittenberger-de Groot AC, Te Velthuis AJ, Bagowski CP: **The lim domain only protein 7 is important in zebrafish heart development**. *Developmental Dynamics* 2008, **237**:3940–3952.
12. Lacroix M, Toillon RA, Leclercq G: **p53 and breast cancer, an update**. *Endocrine-Related Cancer* 2006, **13**:293–325.
13. Kim SY, Dunn IF, Firestein R, Gupta P, Wardwell L, Repich K, Schinzel AC, Wittner B, Silver SJ, Root DE, Boehm JS, Ramaswamy S, Lander ES, Hahn WC: **CK1e is required for breast cancers dependent on beta-Catenin activity**. *PLOS ONE* 2010, **5**:e8979.
14. Florena AM, Tripodo C, Guarnotta C, Ingrao S, Porcasi R, Martorana A, Lo Bosco G, Cabibi D, Franco V: **Associations between Notch-2, Akt-1 and HER2/neu expression in invasive human breast cancer: a tissue microarray immunophenotypic analysis on 98 patients**. *Pathobiology* 2007, **74**:317–322.
15. Clark J, Edwards S, John M, Flohr P, Gordon T, Maillard K, Giddings I, Brown C, Bagherzadeh A, Campbell C, Shipley J, Wooster R, Cooper CS: **Identification of amplified and expressed genes in breast cancer by comparative hybridization onto microarrays of randomly selected cDNA clones**. *Genes, Chromosomes and Cancer* 2002, **33**:104–114.
16. Dettling M, Gabrielson E, Parmigiani G: **Searching for differentially expressed gene combinations**. *Genome Biology* 2005, **6**:R88.
17. Imai K, Morio T, Zhu Y, Jin Y, Itoh S, Kajiwara M, Yata J, Mizutani S, Ochs HD, Nonoyama S: **Clinical course of patients with WASP gene mutations**. *Blood* 2004, **103**:456–464.
18. Choschzick M, Lebeau A, Marx AH, Tharun L, Terracciano L, Heilenkotter U, Jaenicke F, Bokemeyer C, Simon R, Sauter G, Schwarz J: **Overexpression of cell division cycle 7 homolog is associated with gene amplification frequency in breast cancer**. *Human Pathology* 2010, **41**:358–365.
19. Takaoka A, Yanai H, Kondo S, Duncan G, Negishi H, Mizutani T, Kano S, Honda K, Ohba Y, Mak TW, Taniguchi T: **Integral role of IRF-5 in the gene induction progtamme activated by Toll-like receptors**. *Nature* 2005, **434**:243–249.
20. Fong S, Mounkes L, Liu Y, Maibaum M, Alonzo E, Desprez PY, Thor AD, Kashani-Sabet M, Debs RJ: **Functional identification of distinct sets of antitumor activities mediated by the FKBP gene family**. *PNAS* 2003, **100**:14253–14258.
21. Lu J, Guo H, Treekitkarnmongkol W, Li P, Zhang J, Shi B, Ling C, Zhou X, Chen T, Chiao PJ, Feng X, Seewaldt VL, Muller WJ, Sahin A, Hung MC, Yu D: **14-3-3zeta Cooperates with ErbB2 to promote ductal carcinoma in situ progression to invasive breast cancer by inducing epithelial-mesenchymal transition**. *Cancer Cell* 2009, **16**:195–207.
22. Jang JY, Choi Y, Jeon YK, Kim CW: **Suppression of adenine nucleotide translocase-2 by vector-based siRNA in human breast cancer cells induces apoptosis and inhibits tumor growth in vitro and in vivo**. *Breast Cancer Research* 2008, **10**:R11.
23. Lei KF, Wang YF, Zhu XQ, Lu PC, Sun BS, Jia HL, Ren N, Ye QH, Sun HC, Wang L, Tang ZY, Qin LX: **Identification of MSRA gene on chromosome 8p as a candidate metastasis suppressor for human hepatitis B virus-positive hepatocellular carcinoma**. *BMC Cancer* 2007, **7**:172.
24. Ma XJ, Salunga R, Dahiya S, Wang W, Carney E, Durbecq V, Harris A, Goss P, Sotiriou C, Erlander M, Sgroi D: **A five-gene molecular grade index and HOXB13:IL17BR are complementary prognostic factors in early stage breast cancer**. *Clinical Cancer Research* 2008, **14**:2601–2608.

25. Hernandez-Vargas H, Ballestar E, Carmona-Saez P, von Kobbe C, Banon-Rodrigue I, Esteller M, Moreno-Bueno G, Palacios J: **Transcriptional profiling of MCF7 breast cancer cells in response to 5-Fluorouracil: relationship with cell cycle changes and apoptosis, and identification of novel targets of p53**. *International Journal of Cancer* 2006, **119**:1164–1175.

26. Bertucci F, Finetti P, Rougemont J, Charafe-Jauffret E, Nasser V, Loriod B, Camerlo J, Tagett R, Tarpin C, Houvenaeghel G, Nguyen C, Maraninchi D, Jacquemier J, Houlgatte R, Birnbaum D, Viens P: **Gene expression profiling for molecular characterization of inflammatory breast cancer and prediction of response to chemotherapy**. *Cancer Research* 2004, **64**:8558–8565.

27. Ajiro M, Katagiri T, Ueda K, Nakagawa H, Fukukawa C, Lin ML, Park JH, Nishidate T, Daigo Y, Nakamura Y: **Involvement of RQCD1 overexpression, a novel cancer-testis antigen, in the Akt pathway in breast cancer cells**. *International Journal of Oncology* 2009, **35**:673–681.

28. Sutherland RL, Musgrove EA: **CDK inhibitors as potential breast cancer therapeutics: new evidence for enhanced efficacy in ER+ disease**. *Breast Cancer Research* 2009, **11**:112.

29. Cheng CJ, Lin YC, Tsai MT, Chen CS, Hsieh MC, Chen CL, Yang RB: **SCUBE2 suppresses breast tumor cell proliferation and confers a favorable prognosis in invasive breast cancer**. *Cancer Research* 2009, **69**:3634–3641.

30. Hassan MR, Hossain MM, Bailey J, Macintyre G, Ho JWK, Ramamohanarao K: **A voting approach to identify a small number of highly predictive genes using multiple classifiers**. *BMC Bioinformatics* 2009, **10**:S19.

31. Hu Z, Huang G, Sadanandam A, Gu S, Lenburg ME, Pai M, Bayani N, Blakely EA, Gray JW, Mao JH: **The expression level of HJURP has an independent prognostic impact and predicts the sensitivity to radiotherapy in breast cancer**. *Breast Cancer Research* 2010, **12**:R18.

32. Carboni GL, Gao B, Nishizaki M, Xu K, Minna JD, Roth JA, Ji L: **CACNA2D2-mediated apoptosis in NSCLC cells is associated with alterations of the intracellular calcium signaling and disruption of mitochondria membrane integrity**. *Oncogene* 2003, **22**:615–626.

33. **Genes-to-Systems Breast Cancer Database** [http://www.itb.cnr.it/breastcancer/php/browse.php].

34. Graveel CR, DeGroot JD, Su Y, Koeman J, Dykema K, Leung S, Snider J, Davies SR, Swiatek PJ, Cottingham S, Watson MA, Ellis MJ, Sigler RE, Furge KA, Vande Woude GF: **Met induces diverse mammary carcinomas in mice and is associated with human basal breast cancer**. *PNAS* 2009, **106**(31):12909–12914.

35. Parker BS, Ciocca DR, Bidwell BN, Gago FE, Fanelli MA, George J, Slavin JL, Moller A, Steel R, Pouliot N, Eckhardt BL, Henderson MA, Anderson RL: **Primary tumour expression of the cysteine cathepsin inhibitor Stefin A inhibits distant metastasis in breast cancer**. *The Journal of Pathology* 2008, **214**:337–346.

36. Martinez V, Kennedy S, Doolan P, Gammell P, Joyce H, Kenny E, Prakash Mehta J, Ryan E, O'Connor R, Crown J, Clynes M, O'Driscoll L: **Drug metabolism-related genes as potential biomarkers: analysis of expression in normal and tumour breast tissue**. *Breast Cancer Research and Treatment* 2008, **110**:521–530.

37. Piva P, Pellegrino E, Mattioli M, Agnelli L, Lombardi L, Boccalatte F, Costa G, Ruggeri BA, Cheng M, Chiarle R, Palestro G, Neri A, Inghirami G: **Functional validation of the anaplastic lymphoma kinase signature identifies CEBPB and Bcl2A1 as critical target genes**. *The Journal of Clinical Investigation* 2006, **116**:3171–3182.

38. Watanuki-Miyauchi R, Kojima Y, Tsurumi H, Hara T, Goto N, Kasahara S, Saio M, Moriwaki H, Takami T: **Expression of survivin and of antigen detected by a novel monoclonal antibody, T332, is associated with outcome of diffuse large B-cell lymphoma and its subtypes**. *Pathology International* 2005, **55**:324–330.

39. Liu L, Zhang M, Zou P: **Expression of PLK1 and survivin in diffuse large B-cell lymphoma**. *Leukemia and Lymphoma* 2007, **48**:2179–2183.

40. Houldsworth J, Petlakh M, Olshen AB, Chaganti RSK: **Pathway activation in large B-cell non-Hodgkin lymphoma cell lines by doxorubicin reveals prognostic markers of in vivo response**. *Leukemia and Lymphoma* 2008, **49**(11):2170–2180.

41. Larsen E, Kleppa L, Meza TJ, Meza-Zepeda LA, Rada C, Castellanos CG, Lien GF, Nesse GJ, Neuberger MS, Laerdahl JK, William Doughty R, Klungland A: **Early-onset lymphoma and extensive embryonic apoptosis in two domain-specific Fen1 mice mutants**. *Cancer Research* 2008, **68**(12):4571–4579.

42. Martinez-Climent JA, Alizadeh AA, Segraves R, Blesa D, Rubio-Moscardo F, Albertson DG, Garcia-Conde J, Dyer MJ, Levy R, Pinkel D, Lossos IS: **Transformation of follicular lymphoma to diffuse large cell lymphoma is associated with a heterogeneous set of DNA copy number and gene expression alterations**. *Blood* 2003, **101**(8):3109–3117.

43. Kidokoro T, Tanikawa C, Furukawa Y, Katagiri T, Nakamura Y, Matsuda K: **CDC20, a potential cancer therapeutic target, is negatively regulated by p53**. *Oncogene* 2008, **27**(11):1562–1571.

44. Davies AJ, Rosenwald A, Wright G, Lee A, Last KW, Weisenburger DD, Chan WC, Delabie J, Braziel RM, Campo E, Gascoyne RD, Jaffe ES, Muller-Hermelink HK, Ott G, Calaminici M, Norton AJ, Goff LK, Fitzgibbon J, Staudt LM, Lister TA: **Transformation of follicular lymphoma to diffuse large B-cell lymphoma proceeds by distinct oncogenic mechanisms**. *British Journal of Haematology* 2007, **136**(2):286–293.

45. Kalmanti M, Kanavaros P, Sakalidou A, Tzardi M, Datseris G, Bolonaki E, Kalmantis T, Kazlaris E, Delides G: **Proliferating cell nuclear antigen (PCNA) expression in childhood lymphomas**. *Acta Morphol Hung* 1992, **40**:215–221.

46. Smith FG, Murray PG, Crocker J: **Correlation between PCNA and AgNOR scores in non-Hodgkin's lymphomas using sequential staining technique**. *Journal of Clinical Pathology* 1993, **46**:28–31.

47. Jankovic D, Gorello P, Liu T, Ehret S, La Starza R, Desjobert C, Baty F, Brutsche M, Jayaraman PS, Santoro A, Mecucci C, Schwaller J: **Leukemogenic mechanisms and targets of a NUP98/HHEX fusion in acute myeloid leukemia**. *Blood* 2008, **111**(12):5672–5682.

48. Lossos IS, Czerwinski DK, Alizadeh AA, Wechser MA, Tibshirani R, Botstein D, Levy R: **Prediction of survival in diffuse large-B-cell lymphoma based on the expression of six genes**. *NEJM* 2004, **350**:1828–1837.

49. He Q, Zhang P, Zou L, Li H, Wang X, Zhou S, Fornander T, Skog S: **Concentration of thymidine kinase 1 in serum (S-TK1) is a more sensitive proliferation marker in human solid tumors than its activity**. *Oncology Reports* 2005, **14**:1013–1019.

50. Gopee NV, Johnson VJ, Sharma RP: **Sodium selenite-induced apoptosis in murine B-lymphoma cells is associated with inhibition of protein kinase C-delta, nuclear factor kappaB, and inhibitor of apoptosis protein**. *Toxicological Sciences* 2004, **78**:204–214.

51. Lee HK, Hsu AK, Sajdak J, Qin J, Pavlidis P: **Coexpression analysis of human genes across many microarray data sets**. *Genome Research* 2004, **14**:1085–1094.

52. Daibata M, Nemoto Y, Bandobashi K, Kotani N, Kuroda M, Tsuchiya M, Okuda H, Takakuwa T, Imai S, Shuin T, Taguchi H: **Promoter hypermethylation of the bone morphogenetic protein-6 gene in malignant lymphoma**. *Clinical Cancer Research* 2007, **13**:3528–3535.

53. Piccaluga PP, Agostinelli C, Califano A, Rossi M, Basso K, Zupo S, Went P, Klein U, Zinzani PL, Baccarani M, Favera RD, Pileri SA: **Gene expression analysis of peripheral T cell lymphoma, unspecified, reveals distinct profiles and new potential therapeutic targets**. *The Journal of Clinical Investigation* 2007, **117**:823–834.
